# Experimental Infection of Reindeer with Jamestown Canyon Virus

**DOI:** 10.3201/eid3012.240757

**Published:** 2024-12

**Authors:** Kayla J. Buhler, John Blake, Heather Fenton, Isaac H. Solomon, Emily Jenkins

**Affiliations:** Inland Norway University of Applied Sciences, Innlandet, Norway (K.J. Buhler); University of Saskatchewan, Saskatoon, Saskatchewan, Canada (K.J. Buhler, E. Jenkins); University of Alaska Fairbanks, Fairbanks, Alaska, USA (J. Blake); Australian Registry of Wildlife Health, Taronga Conservation Society, Mosman, New South Wales, Australia (H. Fenton); Ross University School of Veterinary Medicine, Basseterre, Saint Kitts and Nevis (H. Fenton); Brigham and Women’s Hospital and Harvard Medical School, Boston, Massachusetts, USA (I.H. Solomon)

**Keywords:** Jamestown Canyon virus, arboviruses, California serogroup viruses, reindeer, Alaska, USA, *Suggested citation for this article*: Buhler KJ, Blake J, Fenton H, Solomon IH, Jenkins E. Experimental infection of reindeer with Jamestown Canyon virus. Emerg Infect Dis. 2024 Dec [*date cited*]. https://doi.org/10.3201/eid3012.240757

## Abstract

Seroprevalence of Jamestown Canyon virus in free-ranging caribou in North America is high. We demonstrate serum antibodies and RNA of the virus in blood and tissues of experimentally exposed reindeer with no clinical illness and minimal histopathologic changes. Caribou and reindeer may play a role in emergence and dissemination of vectorborne zoonoses in Arctic regions.

California serogroup viruses (CSVs), including snowshoe hare virus and Jamestown Canyon virus (JCV), are mosquitoborne orthobunyaviruses present throughout northern North America (*1*). Infection typically causes influenza-like symptoms and rarely encephalitis, most commonly in children with snowshoe hare virus and adults with JCV infection (*2*). High CSV exposure has been documented in humans (27% of Alaska residents) and animals (64% of caribou with antibodies to JCV) in the Nearctic (*3*,*4*). Our serologic evidence suggests that caribou may be natural wildlife hosts for JCV, as has been suggested for white-tailed deer in temperate regions (*5*). Caribou and reindeer (*Rangifer tarandus*) play an essential role in northern ecosystems, are declining substantially in many regions, and are culturally significant to Indigenous populations (*6*). We conducted this study to gain more knowledge about the role of caribou in JCV disease ecology. Animal experimentation was approved by the University of Alaska Fairbanks Institutional Animal Care and Use Committee (IACUC 1552224, 1654054, and 1654058) and the University of Saskatchewan Animal Care Committee (20210075 and 20210076).

## The Study

**Figure 1:**
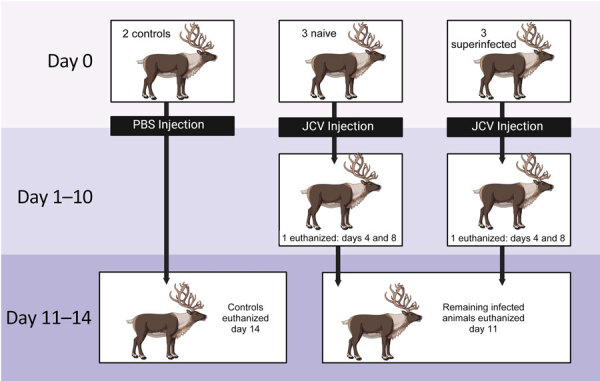
Study design for experimental infection of reindeer with JCV. JCV, Jamestown Canyon virus; PBS, phosphate-buffered saline.

In May and December 2020, we screened 30 reindeer from the Large Animal Research Station (University of Alaska Fairbanks; UAF) for CSV antibodies by using cELISA (*4*,*7*) (Appendix Figure 1). After a summer of insect activity, seroprevalence remained the same among the adults (91%; n = 10/11), but 84% (n = 16/19) of young animals had seroconverted. We housed a control group (1 infected and 1 naive female) in a separate pen from experimentally exposed naive reindeer (2 females, 1 male) and a superinfected group (2 females, 1 male) (Figure 1). Exposed animals received 10^6^ PFU of JCV in 1 mL of sterile phosphate buffered saline (PBS), pH 7.2, administered subcutaneously in the right shoulder. The control group received 1 mL PBS without virus, administered identically.

We disinfected handling facilities daily with a 10% bleach solution and collected rectal temperatures, weights, and jugular blood samples daily. We manually differentiated and quantified leukocytes on EDTA blood smears stained with EASY III (Azer Scientific, https://www.azerscientific.com). We anesthetized animals with intramuscular ketamine (7–10 mg/kg) and xylazine (0.6 mg/kg) before we euthanized them with intravenous pentobarbital (60–80 mg/kg) (Euthasol; Virbac, https://us.virbac.com) (Figure 1). During necropsy, we collected cerebral spinal fluid in red top BD Vacutainer tubes (https://www.bd.com) and placed oral, nasal, and conjunctival swab samples in tubes containing 1.0 mL sterile PBS, stored at −80°C until shipping. We subdivided tissues (liver, kidney, spleen, lung, gonad, brain, spinal cord, quadriceps, heart, muscle under the injection site, tonsils, parotid lymph nodes, retropharyngeal and mesenteric lymph nodes) and stored them frozen (−20°C) and fixed in 10% neutral buffered formalin. We also collected heart blood, chest fluid, abdominal fluid, urine, and feces if present. We disinfected instruments between each animal. We transferred all samples to the Zoonotic Parasite Research Unit (Western College of Veterinary Medicine, Saskatoon, SK, Canada) and stored at −20°C until testing.

We extracted RNA from tissues and fluids by using the RNeasy Mini Kit (QIAGEN, https://www.qiagen.com) and performed reverse transcription PCR with JCV primers and probe (*4*). We considered samples at <30 quantification cycles (Cq) to be positive. Positive controls were gBlocks gene fragments (Integrated DNA Technologies, https://www.idtdna.com) created from the small (S) segment of JCV (GenBank accession no. MN135989.1).

Tissues were trimmed, embedded in paraffin, cut, and stained with hematoxylin and eosin after 30 days in formalin at Prairie Diagnostic Services Inc. (Saskatoon). Slides were read by a blinded, board-certified, veterinary pathologist. Paraffin-embedded sections of brain (cerebrum, cerebellum, and obex), cranial lymph node, tonsil, and a gastrointestinal section with myenteric plexus were sent to Brigham and Women’s Hospital and Harvard Medical School (Boston, MA, USA) for in situ hybridization (ISH) and immunohistochemistry (Appendix Table 1). RNA ISH was performed on a Leica BOND-III system (Leica Biosystems, https://www.leicabiosystems.com) with RNAScope probes V-JCV-LMS-O1 (1187658-C1) (Advanced Cell Diagnostics, https://www.acdbio.com) according to manufacturer protocols and 14 ZZ probes targeting JCV isolate 11497-03; segments tested were 52–814 bp of the S segment (GenBank accession no. EF681845.1), 2–462 bp of the medium (M) segment (accession no. EF687121.1), and 227–337 bp of the large (L) segment (accession no. EF687059.1). We performed immunohistochemistry by using the Leica-BOND-III system with rabbit polyclonal GFAP antibody (1:3,000 dilution; abcam, https://www.abcam.com) and rabbit polyclonal IBA1 antibody (1:100 dilution; FUJIFILM Wako Chemicals Corp., https://www.fujifilm.com). Slides were reviewed by a blinded, board-certified, medical neuropathologist.

**Figure 2:**
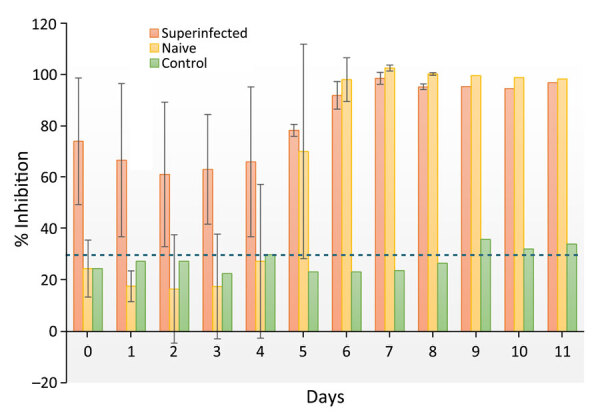
Average antibody response for naive and superinfected groups of reindeer experimentally infected with Jamestown Canyon virus. The titer of the naive control animal is provided for comparison. SD (error bars) are included when groups contained >1 animal. The dashed blue line indicates the positive cutoff value of the competitive ELISA (30% inhibition).

Throughout the experiment, we observed no changes in behavior or body temperature of the reindeer (Appendix Table 2). The naive exposed group seroconverted on postinfection day 5, and antibody titers for all exposed reindeer peaked at postinfection day 7 (Figure 2). Lymphocytes were consistently elevated for reindeer in the naive and superinfected exposed groups but not in the control group. Viral RNA was detected in blood on days 1 (Cq = 13.80; naive animal) and 5 (Cq = 10.08; superinfected animal). Viral RNA was detected in chest fluid (Cq = 11.47), urine (Cq = 20.26), spleen (Cq = 22.04), parotid lymph node (Cq = 16.26), obex (Cq = 28.03), and uterus (Cq = 11.66) from exposed but not control animals (Appendix Table 1).

**Figure 3:**
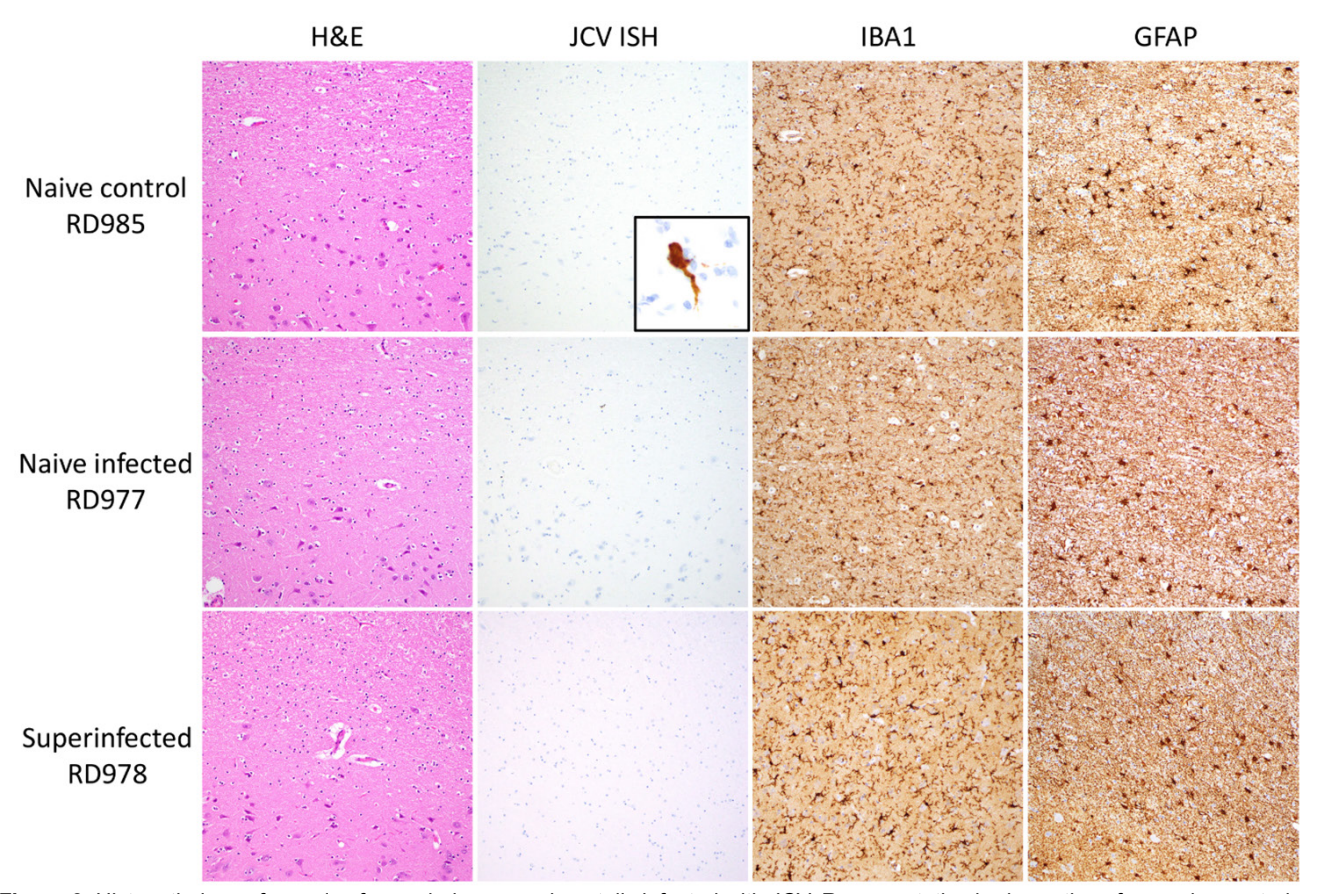
Histopathology of samples from reindeer experimentally infected with JCV. Representative brain sections from naive control (RD985), naive infected (RD977), and superinfected (RD978) animals shows H&E-stained sections of cerebral cortex and subcortical white matter with minimal diagnostic abnormalities, negative JCV RNA ISH staining, scattered IBA1-positive microglia/macrophages, and mild-to-moderate astrocytosis highlighted by GFAP immunohistochemistry. JCV ISH naive control inset panel demonstrates positive cytoplasmic staining in a cortical neuron from a positive control case of fatal JCV encephalitis in a human. All images taken with 20× objective with exception of the JCV ISH inset, taken with 60× objective. GFAP, glial fibrillary acidic protein; H&E, hematoxylin and eosin; IBA, allograft inflammatory factor 1; ISH, in situ hybridization; JCV, Jamestown Canyon virus.

Histopathology revealed subtle, nonspecific neuronal changes in the brains of all animals except the naive control. Mild congestion and lymphoplasmacytic inflammation were detected in kidneys (n = 8), urinary bladder (n = 1, naive control), liver (n = 4, including previously infected control), and conjunctiva (n = 1, previously infected control). In situ hybridization did not reveal viral RNA within lesions. Immunohistochemistry for GFAP (glial fibrillary acidic protein) showed mild to moderate astrogliosis and for IBA1 (allograft inflammatory factor 1) showed mild microgliosis in brain sections (Figure 3).

## Conclusions

We demonstrate JCV experimental infection in reindeer with no clinical signs of illness and minimal, nonspecific histological changes, similar to what has been reported for other animal arbovirus infections (*8*). Our results are consistent with those of a previous experiment in which white-tailed deer were infected with JCV and were viremic for 2–4 days but showed no clinical illness (*9*). We detected RNA in blood in the chest cavity of 1 animal at postinfection day 11, suggesting that that reindeer may be viremic for longer. The high rate of seroconversion in the naturally exposed herd of 30 reindeer suggests that reindeer and caribou may play a role in the ecology of CSVs in northern ecosystems, especially given that most young animals seroconverted within a single summer (11%–84%). Further studies focusing on isolation of live virus from experimentally infected animals in addition to vector competence would provide more information about the potential for reindeer to be maintenance hosts of the virus (*10*).

In humans, neuroinvasive disease is a strong component of JCV disease (*11*). We note that genetic material was found in the obex of 1 reindeer, suggesting potential infection, but ISH and immunohistochemistry results did not demonstrate neuronal invasion, despite a potential glial response. Although JCV was detected in the uterus, the implications for potential vertical spread or other effects are unknown. Single-stranded RNA viruses are not stable in the environment, and multiple freeze–thaw events probably affected genetic detection of virus in our study (*12*).

Our detection of JCV in blood and tissues of experimentally exposed reindeer, together with high seroprevalence in free-ranging animals in North America, suggests that *Rangifer* spp. (caribou and reindeer) could play a role in disease ecology of JCV in Arctic and sub-Arctic North America (*4*). 

Appendix Supplemental results for study of experimental infection of reindeer with Jamestown Canyon virus.
