# Supplementary material for: Experimental Infection of Reindeer with Jamestown Canyon Virus
Source: Emerg Infect Dis. 2024 Dec;30(12):2664–8. doi: 10.3201/eid3012.240757 (PMC11616646; doi:10.3201/eid3012.240757)
Supplement: Appendix — Supplemental results for study of experimental infection of reindeer with Jamestown Canyon virus. [file 24-0757-Techapp-s1.pdf]

Article DOI: https://doi.org/10.3201/eid3012.240757

EID cannot ensure accessibility for supplementary materials supplied by authors. Readers who have difficulty accessing supplementary content should contact the authors for assistance.

Experimental Infection of Caribou with Jamestown Canyon Virus

Appendix

Appendix Table 1. RT-PCR results from tissues and fluids collected from reindeer during the experimental infection. Quantification cycles (Cq) are provided and only values ≤ 30 were considered positive. Negatives are indicated by a N.

Sample (ID)	Naïve Control (RD985)	Superinfected Control (RD989)	Superinfected (RD984)	Naïve (RD611)	Naïve (RD977)	Superinfected (RD978)	Superinfected (RD981)	Naïve (RD983)
Collection date post infection	Day 14	Day 14	Day 11	Day 11	Day 8	Day 8	Day 4	Day 4
Conjunctival Swab	N	N	N	N	N	N	N	N
Oral Swab	N	N	N	N	N	N	N	N
Nasal Swab	N	N	N	N	N	N	N	N
CSF	N	N	N	N	N	N	N	N
Liver	N	N	N	N	N	N	N	N
Left Kidney	N	N	N	N	N	N	N	N
Right Kidney	N	N	N	N	N	N	N	N
Quad	N	N	N	N	N	N	N	N
Spleen	N	N	N	22.04	N	N	N	N
Right Cranial Lung	N	N	N	N	N	N	N	N
Right Caudal Lung	N	N	N	N	N	N	N	N
Left Cranial Lung	N	N	N	N	N	N	N	N
Left Caudal Lung	N	N	N	N	N	N	N	N
Heart	N	N	N	N	N	N	N	N
Muscle Under Injection	N	N	N	N	N	N	N	N
Mesenteric Lymph Node	N	N	N	N	NA	N	N	N
Testes or Uterus	N	N	N	N	N	N	11.66	N
Heart Blood	N	N	N	N	N	N	N	N
Chest Fluid	N	N	11.47	N	N	N	N	N
Urine	N	N	N	N	N	20.26	N	N
Feces	N	N	N	N	N	N	N	N
Retropharyngea-l Lymph Node	N	N	N	N	N	N	N	N
Parotid Lymph Node	N	N	N	N	N	16.26	N	N
Tonsil	N	N	N	N	N	N	N	N
Obex	N	N	28.03	N	N	N	N	N
Cerebellum	N	N	N	N	N	N	N	N
Cerebrum	N	N	N	N	N	N	N	N
Spinal Cord	N	N	N	N	N	N	N	N
Abdominal Fluid	N	N	N	N	N	N	N	N
Blood Day 0	N	N	N	N	N	N	N	N
Blood Day 1	N	N	N	N	13.80	N	N	N
Blood Day 2	N	N	N	N	N	N	N	N
Blood Day 3	N	N	N	N	N	N	N	N
Blood Day 4	N	N	N	N	N	N	N	N
Blood Day 5	N	N	N	N	N	10.08	N	N
Blood Day 6	N	N	N	N	N	N	N	N
Blood Day 7	N	N	N	N	N	N	N	N
Blood Day 8	N	N	N	N	N	N	N	N
Blood Day 9	N	N	N	N	N	N	N	N
Blood Day 10	N	N	N	N	N	N	N	N
Blood Day 11	N	N	N	N	N	N	N	N

Appendix Table 2. Weight and temperature of animals included in the experimental infection trial.

		Day 0	1	2	3	4	5	6	7	8	9	10	11
Group	ID	Weight											
Control*	RD989	106.8	105.4	107.8	105.2	105.8	106	106.6	106.2	105.2	104	103	106.8
Control**	RD985	83.8	79.2	79.8	80.6	81.8	80.6	81.4	82.2	83	82.4	80.4	83.8
Naïve	RD983	97.4	98.8	99.4	98.8	98.8	EUTH	EUTH	EUTH	EUTH	EUTH	EUTH	EUTH
Naïve	RD977	140.8	140	141	139.2	138.4	138	137.4	135.4	136	EUTH	EUTH	EUTH
Naïve	RD611	119.8	119.6	119.8	120.4	119	119	118.6	118.4	117.8	118.2	117.8	119.8
Superinfected	RD981	95.2	93.4	94.4	93.4	92.8	EUTH	EUTH	EUTH	EUTH	EUTH	EUTH	EUTH
Superinfected	RD978	120	117.4	117.6	116.8	116.8	115	114.8	114.2	113.6	EUTH	EUTH	EUTH
Superinfected	RD984	96	97.2	98.2	98	97	97.4	97.2	96.2	95	95.2	94.6	96
		Temperature (°C)											
Control*	RD989	38.7	38.8	38.9	38.8	38.8	38.6	38.9	38.4	38.7	39	38.9	NA
Control**	RD985	38.8	38.8	38.8	38.9	38.7	38.6	39.1	38.7	39.1	38.9	38.9	NA
Naïve	RD983	38.6	38.7	38.6	38.7	38.3	EUTH	EUTH	EUTH	EUTH	EUTH	EUTH	EUTH
Naïve	RD977	39.3	39.3	39.4	39.1	38.8	38.3	39.4	39.3	39.2	EUTH	EUTH	EUTH
Naïve	RD611	38.5	38.5	38.3	38.5	37.9	38.7	38.9	38.6	38.5	38.4	38.6	NA
Superinfected	RD981	38.9	39.4	38.9	39.2	39	EUTH	EUTH	EUTH	EUTH	EUTH	EUTH	EUTH
Superinfected	RD978	39.2	38.7	38.7	38.7	38.8	39.3	39.1	39.1	39.2	EUTH	EUTH	EUTH
Superinfected	RD984	38.9	38.7	38.7	38.8	38.5	38.8	39	38.7	39	39.1	39	NA

* Control animal with positive antibody titer prior to the study; ** Naïve control animal; EUTH = euthanized; NA= not available.

Appendix Table 3. Differential white blood cell counts (per 100 cells) for control, naïve and superinfected groups of reindeer.

Differential white blood cell count (per 100 cells)													
Eosinophils													
Group	ID	Day 0	1	2	3	4	5	6	7	8	9	10	11
Control*	RD989	NA	3	2	2	5	2	4	3	2	3	4	4
Control**	RD985	NA	2	1	3	3	5	3	4	3	2	3	6
Naïve	RD983	NA	4	1	4	3	EUTH	EUTH	EUTH	EUTH	EUTH	EUTH	EUTH
Naïve	RD977	NA	5	4	8	8	7	5	8	10	EUTH	EUTH	EUTH
Naïve	RD611	NA	3	3	2	3	2	4	3	4	3	4	3
Superinfected	RD981	NA	3	6	3	2	EUTH	EUTH	EUTH	EUTH	EUTH	EUTH	EUTH
Superinfected	RD978	NA	2	1	5	3	5	6	2	2	EUTH	EUTH	EUTH
Superinfected	RD984	NA	3	4	5	5	7	3	6	6	4	3	7
Lymphocytes													
Control*	RD989	NA	65	66	53	56	58	66	65	62	55	61	56
Control**	RD985	NA	53	64	53	45	48	57	50	65	59	41	50
Naïve	RD983	NA	64	64	63	66	EUTH	EUTH	EUTH	EUTH	EUTH	EUTH	EUTH
Naïve	RD977	NA	65	70	62	56	65	55	66	66	EUTH	EUTH	EUTH
Naïve	RD611	NA	58	71	64	71	71	71	73	66	64	63	51
Superinfected	RD981	NA	65	54	59	58	EUTH	EUTH	EUTH	EUTH	EUTH	EUTH	EUTH
Superinfected	RD978	NA	66	64	63	65	61	73	70	69	EUTH	EUTH	EUTH
Superinfected	RD984	NA	77	72	74	66	72	67	70	70	69	71	67
Monocytes													
Control*	RD989	NA	2	1	1	1	2	2	1	2	1	2	2
Control**	RD985	NA	1	1	1	3	1	1	2	2	0	2	2
Naïve	RD983	NA	2	1	2	1	EUTH	EUTH	EUTH	EUTH	EUTH	EUTH	EUTH
Naïve	RD977	NA	3	2	1	0	2	1	2	4	EUTH	EUTH	EUTH
Naïve	RD611	NA	2	2	4	1	1	1	1	0	1	0	2
Superinfected	RD981	NA	3	3	3	3	EUTH	EUTH	EUTH	EUTH	EUTH	EUTH	EUTH
Superinfected	RD978	NA	2	2	1	0	0	0	0	1	EUTH	EUTH	EUTH
Superinfected	RD984	NA	2	2	2	1	1	1	1	3	2	1	2
Neutrophils													
Control*	RD989	NA	30	30	44	37	37	28	30	34	41	34	39
Control**	RD985	NA	44	34	42	49	46	40	43	34	45	54	42
Naïve	RD983	NA	30	34	29	30	EUTH	EUTH	EUTH	EUTH	EUTH	EUTH	EUTH
Naïve	RD977	NA	26	24	28	35	26	39	24	20	EUTH	EUTH	EUTH
Naïve	RD611	NA	38	24	31	25	27	24	27	30	33	32	44
Superinfected	RD981	NA	30	38	39	37	EUTH	EUTH	EUTH	EUTH	EUTH	EUTH	EUTH
Superinfected	RD978	NA	31	33	31	33	34	21	28	28	EUTH	EUTH	EUTH
Superinfected	RD984	NA	18	21	18	28	20	28	22	21	25	25	24

* Control animal with positive antibody titer prior to the study; ** Naïve control animal; EUTH = euthanized; NA= not available.

$$\% \text{ Inhibition} = 100 - [(TS - B) / (CS - B)] \times 100$$

TS = Mean optical density of test serum
CS = Mean optical density of control serum
B = Background optical density

Appendix Figure. Calculation of percent inhibition for cELISA.
